# Tailored knowledge distillation with automated loss function learning

**DOI:** 10.1371/journal.pone.0325599

**Published:** 2025-06-11

**Authors:** Sheng Ran, Tao Huang, Wuyue Yang

**Affiliations:** 1 Institute of Statistics and Big Data, Renmin University of China, Beijing, China; 2 Beijing Institute of Mathematical Sciences and Applications (BIMSA), Beijing, China; 3 The University of Sydney, Darlington, New South Wales, Australia; Jiangsu Open University, CHINA

## Abstract

Knowledge Distillation (KD) is one of the most effective and widely used methods for model compression of large models. It has achieved significant success with the meticulous development of distillation losses. However, most state-of-the-art KD losses are manually crafted and task-specific, raising questions about their contribution to distillation efficacy. This paper unveils Learnable Knowledge Distillation (LKD), a novel approach that autonomously learns adaptive, performance-driven distillation losses. LKD revolutionizes KD by employing a bi-level optimization strategy and an iterative optimization that differentiably learns distillation losses aligned with the students’ validation loss. Building upon our proposed generic loss networks for logits and intermediate features, we derive a dynamic optimization strategy to adjust losses based on the student models’ changing states for enhanced performance and adaptability. Additionally, for a more robust loss, we introduce a uniform sampling of diverse previously-trained student models to train the loss with various convergence rates of predictions. With the more universally adaptable distillation framework of LKD, we conduct experiments on various datasets such as CIFAR and ImageNet, demonstrating our superior performance without the need for task-specific adjustments. For example, our LKD achieves 73.62% accuracy with the MobileNet model on ImageNet, significantly surpassing our KD baseline by 2.94%.

## 1 Introduction

Over the past decade, artificial intelligence (AI) has undergone unprecedented advancements, leading to increasingly complex and expansive models. As these models grow in size and complexity, it becomes critically important to maintain their high performance while also making them more lightweight and efficient. The rapid progress in deep learning, particularly in domains such as computer vision and natural language processing, has driven an exponential increase in the size and complexity of Deep Neural Networks (DNNs). Notable examples include models like ViT-Huge, with nearly 1 billion parameters [1], and the GPT series, which has expanded from 110 million to 175 billion parameters [2–4]. However, deploying these large-scale models on resource-constrained devices presents significant challenges, underscoring the need for advanced model compression techniques [5–12].

Knowledge Distillation (KD), as introduced by Hinton *et al*. [6], offers a viable strategy for condensing large models by transferring knowledge from a complex “teacher” model to a simpler “student” model. This approach enables the student to approximate the teacher’s output with similar accuracy, enhancing inference speed and reducing memory usage [13, 14]. However, the disparity in capacity and architecture between teacher and student models poses a challenge in fully replicating the teacher’s performance, highlighting the importance of innovative knowledge transfer methods in KD. Early methods utilized KL divergence with temperature scaling [6] and attention mechanisms with MSE loss for feature alignment [15], while recent advancements like DIST [16] incorporate feature-based and relation-based distillation through Pearson correlation-based loss functions, reflecting ongoing efforts to optimize KD processes.

However, existing KD losses, primarily derived from human intuition, may not always align perfectly with distillation performance. This misalignment suggests that the efficacy of such losses can vary significantly across different tasks and datasets, underscoring the absence of a universal solution. For example, losses optimized for specific applications like object detection might not translate well to other tasks, such as image classification. Additionally, even within the same task, adjustments like temperature settings in KD losses necessitate customization for optimal performance across diverse datasets, as evidenced by differing requirements for CIFAR and ImageNet. This highlights the need for a shift towards methodologies that autonomously optimize performance, minimizing dependence on manual, intuition-driven loss design. Such a shift encourages the investigation of adaptive, performance-focused loss function development, aiming to overcome the constraints of traditional, manually crafted losses.

**Fig 1:**
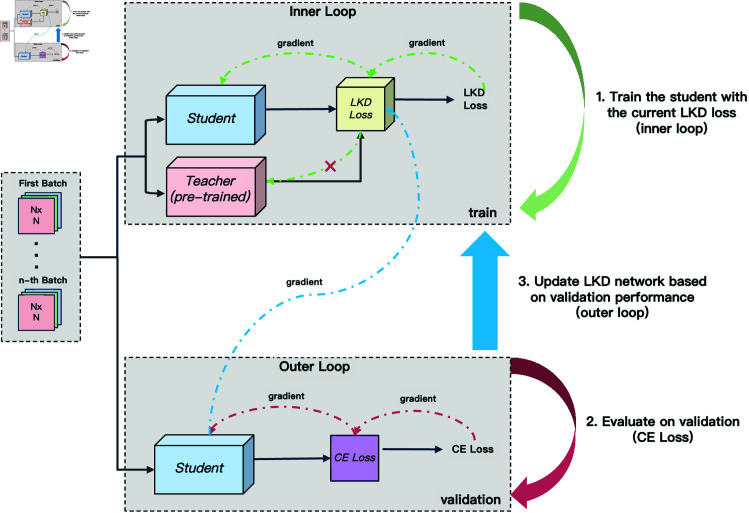
Training pipeline of LKD. The LKD training procedure follows a bi-level optimization scheme with an inner loop for student training and an outer loop for loss network updates. In the inner loop, we train the student model using the current LKD loss, guided by the pre-trained teacher model, and record some of the iterations’ student model parameters. In the outer loop, we evaluate the student model on a validation set using the CE loss, then update the LKD network parameters based on the validation gradients.

This paper introduces Learnable Knowledge Distillation (LKD), a novel approach for creating adaptive, performance-oriented KD losses. LKD treats the development of distillation loss as a bi-level optimization problem as illustrated in Fig 1: the inner level focuses on training the student model using the distillation loss, while the outer level seeks to optimize the loss itself. We propose two types of loss networks for logits and intermediate features, utilizing an approximation method for bi-level optimization that alternates between optimizing the student model and updating the LKD loss based on validation performance. Moreover, a dynamic optimization strategy is integrated, combining traditional KD and LKD training to facilitate immediate adjustments according to the model’s evolving state, ensuring more effective distillation guidance. To enhance the robustness of the loss network, we employ a uniform sampling strategy that selects from previously trained students with varying convergence rates, ensuring the loss’s adaptability to different predictions.

The key contributions of this paper are summarized as follows:

- We introduce an innovative LKD approach, autonomously optimized through a unique training strategy and KD loss framework.
- Our LKD framework incorporates three novel technical advances: (a) A dynamic LKD-Loss model for adaptive loss learning, generating KD loss values responsive to varying inputs and model states; (b) A differentiable iterative loss learning mechanism that leverages validation performance to optimize the loss network; (c) A strategy that uniformly selects historical student models to prevent overfitting and enhance the learning process’s robustness.
- We conduct extensive experiments on ImageNet and CIFAR datasets, and the results demonstrate our versatility and superiority. For example, our LKD method elevates the average top-1 accuracy to 73.04% on the ImageNet dataset, underscoring its exceptional efficacy over alternative KD approaches.

## 2 Related work

### 2.1 Knowledge distillation

The concept of KD was firstly proposed by [6], which utilizes a temperature-scaled softmax to soften teacher outputs, employing Kullback-Leibler (KL) divergence for combining soft and hard labels, foundational in KD methodology. FitNet [17] and AT [18] advance KD through intermediate layer knowledge and attention mechanisms, respectively, using Mean Square Error (MSE) Loss for feature alignment. NST [19] introduces a kernel-based loss for minimizing feature distribution discrepancies, while FSP [20] focuses on capturing inter-layer feature relationships through the FSP matrix. PKT [21] and FT [22] propose probabilistic knowledge transfer and factor transfer methods, enhancing cross-modal knowledge transfer and model output encoding, respectively. SP [23] and CC [24] explore relational knowledge distillation by minimizing variances in activation patterns, while VID [25] uses mutual information to maximize knowledge assimilation. RKD [26] introduces distance and angle-wise loss functions for capturing data point relationships, and AB [27] focuses on activation boundaries for deeper knowledge assimilation. CRD [28] integrates contrastive learning for distinguishing between teacher and student feature patterns. Despite advances, bridging the representation gap remains challenging, with multi-assistant and student-friendly teacher models [29–33] and DIST’s correlation-based loss [16] offering potential solutions. However, all the above-complicated loss functions are manually designed, and it is challenging to intuitively design a novel loss function that performs better than those methods. Therefore, for a better performance-oriented loss, this paper aims to automate the design of distillation losses by using the distillation performance as an objective.

### 2.2 Adaptive knowledge distillation loss

The adaptive KD loss concept aims to refine the traditional KD process to enhance student model performance by overcoming the constraints of static temperature scaling and stringent matching protocols. Unlike standard KD losses, adaptive KD offers a tailored approach, adjusting the distillation loss to better suit the characteristics of teacher and student models, their training progress, and the nature of input samples, among other factors. For example, ATKD [34] suggests dynamically modifying the softmax temperature parameter based on the sharpness gap between teacher and student logits; MKD [35] employs meta-learning to determine an optimal temperature; TTM [36] drops the temperature scaling on the student’s side and introducing Rényi entropy as an additional regularization term to improve the generalization of the student; SRKD [37] utilizes insights from multiple teachers, personalizing the distillation process based on the inter-teacher relational knowledge, offering a nuanced conduit for knowledge transfer; ATD [38] applies variable temperature settings to more efficiently mine and convey knowledge from the teacher to the student, especially targeting samples where the student model encounters difficulties. Nonetheless, these adaptive KD loss methodologies often rely on heuristic, manually-designed mechanisms to tweak traditional KD loss such as KL divergence, without concrete evidence that these adjustments directly correlate with distillation efficiency. Furthermore, their modifications to the traditional loss function, such as adjustments to the temperature parameter or loss weight, offer limited scope for enhancing performance or learning an optimized, performance-oriented loss function. This highlights a critical area for further research and development, aiming to devise more sophisticated adaptive mechanisms that can dynamically and more accurately align the distillation process with the desired performance outcomes.

In supplementary material, we included a detailed discussion of the challenges in automatically designing KD losses with comparisons of current loss designs, to showcase the benefit of the proposed method.

## 3 Revisiting knowledge distillation

In the domain of KD, the fundamental challenge resides in accurately quantifying and subsequently minimizing the divergence between the predictive distributions of the teacher and student models. The KL divergence stands as a critical metric in this endeavor, prized for its efficacy in measuring the disparity in information content across probability distributions. Such divergence furnishes a structured methodology enabling the student model to closely emulate the teacher’s output distribution.

In the formal definition, given the student’s logits $Z^{\left(s\right)}\in {\mathbb{R}}^{B\times C}$ and the teacher’s logits $Z^{\left(t\right)}\in {\mathbb{R}}^{B\times C}$, both of dimension *B* × *C* for batch size *B* and number of classes *C*, the original knowledge distillation loss as introduced by [6] is articulated as follows:

$${ℒ}_{KD}:=\frac{1}{B}\sum_{i=1}^{B}KL\left(Y_{i,:}^{\left(t\right)},Y_{i,:}^{\left(s\right)}\right)=\frac{1}{B}\sum_{i=1}^{B}\sum_{j=1}^{C}Y_{i,j}^{\left(t\right)}\log\left(\frac{Y_{i,j}^{\left(t\right)}}{Y_{i,j}^{\left(s\right)}}\right),$$ (1)

where KL denotes the Kullback-Leibler divergence, with the student’s and teacher’s softmax-transformed logits given by the following equations:

$$Y_{i,:}^{\left(s\right)}=\mathrm{softmax}\left(Z_{i,:}^{\left(s\right)}/\tau \right),\;Y_{i,:}^{\left(t\right)}=\mathrm{softmax}\left(Z_{i,:}^{\left(t\right)}/\tau \right)$$ (2)

In the equations above, $Y_{i,:}^{\left(s\right)}$ and $Y_{i,:}^{\left(t\right)}$ represent the probability distributions of the student and teacher models, respectively, with τ to adjust the distribution’s entropy, enabling a smoother probability distribution that more richly captures the relationships between different classes.

Alongside leveraging the soft targets from the teacher as indicated in Eq 1, the knowledge distillation process as outlined by KD [6] advocates for the simultaneous training of the student model using true labels. This dual-faceted training regime encompasses both the original classification loss ${ℒ}_{cls}$ and the knowledge distillation loss ${ℒ}_{KD}$, thus:

$${ℒ}_{tr}=\alpha {ℒ}_{cls}+\beta {ℒ}_{KD}$$ (3)

where α and β are coefficients to balance the two types of loss, and ${ℒ}_{cls}$ corresponds to the CE loss, which quantifies the discrepancy between the student network’s predictions and the actual ground-truth labels.

## 4 Learnable KD loss

### 4.1 Differentiable optimization of loss

Building upon the discussion in Section 3, it is established that an effective distillation loss function should integrate inputs from both the teacher’s features, $Y^{\left(t\right)}$, and the student’s features, $Y^{\left(s\right)}$, resulting in output within the domain of ${\mathbb{R}}^{1}$. Drawing inspiration from the concept of surrogate loss learning as discussed by [39], this study introduces a novel construct: the LKD loss. This approach conceptualizes the distillation loss as a function computable via a neural network, parameterized by a set of learnable variables, θ. This function, represented as ${ℒ}_{LKD}(Y^{\left(t\right)},Y^{\left(s\right)};\theta )$, underscores a pivotal shift towards a model where the distillation loss itself is subject to optimization and learning. The detailed architectures underlying our LKD loss will be expounded upon in Section 4.3.

Given that all computations within our neural network framework are differentiable, the optimization of the loss function can be effectively performed via gradient descent. To accurately describe the process of differentiable optimization, it is essential to clarify the primary objective in refining the distillation loss. The central focus lies in enhancing the performance of the student model after the distillation process. Specifically, the effectiveness of a distillation loss is determined by its capacity to substantially improve the student model’s accuracy over multiple training iterations.

A direct approach to optimizing the loss function involves applying the loss to train the student model and subsequently evaluating the improvement in the student’s performance as the criterion for loss optimization. This approach positions the optimization of the parameters, θ, as a bi-level optimization problem [40, 41], which can be formalized as follows:

$$\min_{\theta }\;{ℒ}_{val}\left(\omega^{*}(\theta )\right)$$ (4)

$$s.t.\;\omega^{*}(\theta )={argmin}_{\omega }{ℒ}_{LKD}\left(Y^{\left(t\right)},Y^{\left(s\right)};\theta \right).$$ (5)

In this formulation, the inner loop adheres to the conventional principles of knowledge distillation, wherein the student model, parameterized by ω, is optimized against the LKD loss, ${ℒ}_{LKD}$. The outer loop is designed to determine the optimal set of parameters, θ, for the loss function itself, with the aim of achieving the highest possible distillation efficacy, as evidenced by minimal validation error. It is important to note that the validation loss can be employed to optimize the LKD loss parameters, θ, as demonstrated by the following equation:

$$\frac{\partial {ℒ}_{val}}{\partial \theta }=\frac{\partial {ℒ}_{val}}{\partial \omega }\times \frac{\partial \omega }{\partial \theta }$$ (6)

The gradient of the validation loss with respect to θ is computed using the chain rule of derivatives. Specifically, $\frac{\partial {ℒ}_{val}}{\partial \omega }$ is obtained during the validation stage, while $\frac{\partial \omega }{\partial \theta }$ is derived during the training stage using automatic differentiation techniques.

In this context, the cross-entropy (CE) loss is utilized as a measure of validation loss to evaluate the performance of the student model. This approach ensures that the optimization of the distillation process is directly linked to improvements in the student model’s generalization capabilities.

Given the prohibitive complexity associated with directly engaging in the bi-level optimization process, this study introduces a pragmatic approximation strategy that simplifies the iterative optimization of both the student model and the distillation loss, as shown in Fig 1. This streamlined approach, detailed in Algorithm 1, alternates between optimizing the student model and refining the distillation loss, effectively balancing the computational demands.

**Algorithm 1. Differentiable learning of distillation loss.**

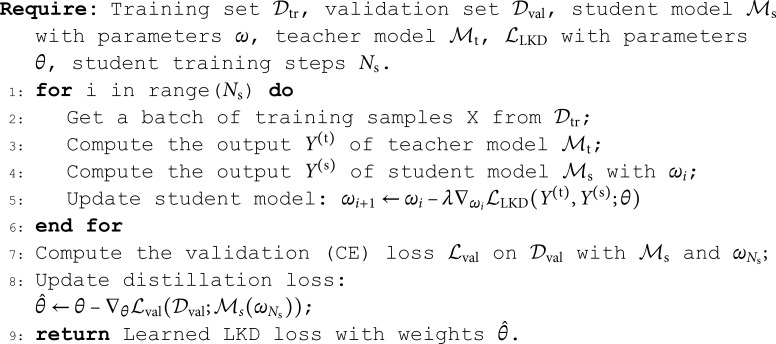



During each iteration of distillation loss learning, the process begins with the selection of a student model for KD training. Utilizing a pre-trained teacher model, the learnable distillation loss then guides the training of the student model over $N_{s}$ steps. In each step, the student weights are updated by the gradients w.r.t. the LKD loss, i.e.,

$$\omega_{i+1}=\omega_{i}-\lambda \nabla_{\omega_{i}}{ℒ}_{LKD}\left(Y^{\left(t\right)},Y^{\left(s\right)};\theta \right).$$ (7)

Crucially, the dependency of the student model’s weights on the distillation loss function allows for the calculation of gradients of the loss function’s parameters relative to the validation loss observed in the student model. Consequently, we can obtain the optimization rule of the LKD loss weights θ as follows:

$$\hat{\theta }=\theta -\eta \nabla_{\theta }{ℒ}_{val}\left({𝒟}_{val};{ℳ}_{s}(\omega_{N_{s}})\right).$$ (8)

The gradient descent for minimizing this validation loss directly contributes to the refinement of the distillation loss, thereby enhancing the student model’s performance with a more focused and efficient learning objective. In other words, by descending ${ℒ}_{val}$ in Equation 8, the new θ is expected to obtain lower validation loss on the distilled student model, and thus inferring better distillation performance.

**Two-stage KD training.** Taking the additional learning of LKD loss into account, the overall KD training of the student network can be separated into two stages: (1) the first stage learns distillation loss by sampling various student models and weights; (2) the second stage fixes the learned distillation loss and uses it as the conventional distillation loss to train the student.

### 4.2 Dynamic optimization of LKD

**Limitations of two-stage optimization.** The traditional two-stage optimization in knowledge distillation, which applies distillation loss statically across all training phases, requires the student model to be pre-trained and to maintain its intermediate weights, leading to significant computational costs. This method, separating loss learning from student training, also faces challenges in developing an effective distillation loss due to varying student states and learning distributions. To address these issues, our work introduces a dynamic optimization strategy that combines distillation loss learning and student model training into a single stage. Starting with a randomly initialized distillation loss, it updates the loss based on the student model’s state after each training epoch, ensuring the loss is continuously aligned with the student’s learning progress. This approach reduces the computational overhead by eliminating the need for repeated training of the student model and closely aligns with the simpler, cost-effective conventional knowledge distillation methods without sacrificing performance.

**Uniform sampling of historical students.** Our empirical investigations revealed that an exclusive focus on the most recent weights of the student model imposes undue constraints, detrimentally impacting the generalization capability of the distillation loss. To mitigate this issue, we advocate for a balanced approach that involves uniformly sampling from the spectrum of weights obtained in previous training epochs of the student model. This strategy is designed to prevent the LKD loss from overfitting to the nuances of the latest model iteration, thereby fostering greater stability throughout the training process. By diversifying the weight samples considered in the optimization of the LKD loss, we enhance its ability to generalize across various stages of the student model’s learning trajectory, promoting a more robust and effective knowledge distillation.

**Gaussian sampling of steps *N***_***s***_. Within the framework of the LKD learning process, the determination of the student model’s training steps, denoted as *N*_*s*_, employs a strategic Gaussian sampling technique. This methodological choice introduces a dynamic element to the progression of the LKD optimization process. By setting the mean of the Gaussian distribution to 25, we ensure that, with a 95% confidence level, the sampled number of steps will predominantly fall within the range of 20 to 30 steps. Such adaptive Gaussian sampling serves as a foundational component of the dynamic optimization strategy in LKD, significantly contributing to the overall effectiveness of the knowledge distillation.

The comprehensive procedure adopted in our method is delineated in Algorithm 2.

**Algorithm 2. KD with dynamic learning of LKD.**

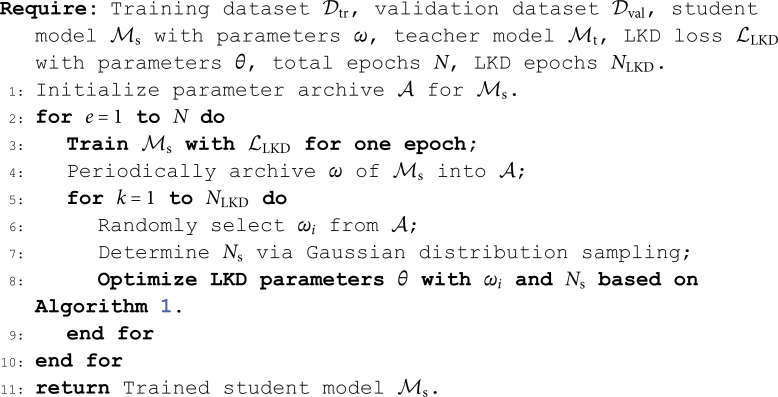



### 4.3 Loss network design

In this paper, to sufficiently validate the efficacy of the proposed method and enhance the performance, we explored LKD losses on predicted logits and intermediate features, namely logits-level LKD loss and feature-level LKD loss.

**Fig 2:**
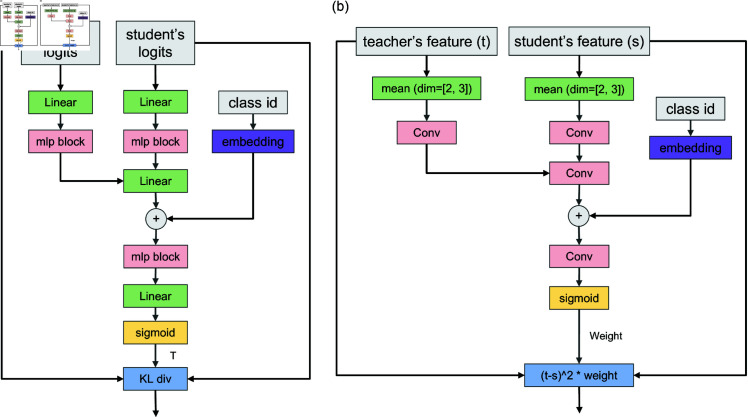
Architectures of LKD losses.

**Logits-level LKD loss.** Central to the LKD framework, the logits-level LKD loss network is a crucial component designed to minimize the disparity in probability distributions between the softened outputs of the student and teacher models, thereby optimizing the distillation process for enhanced efficacy. This process is illustrated in Fig 2, which outlines the architecture and operational dynamics of the network.

Initially, logits from both the student and teacher models undergo processing through distinct linear layers, followed by a sequence of multilayer perceptron (mlp) blocks that feature a combination of linear layers and activation functions. This preprocessing serves to condition the logits for subsequent integration, which is facilitated by an additional linear layer. At this juncture, a class-specific embedding, derived from the ‘class id’, is introduced to the network, embedding class-tailored information directly into the distillation framework.

The network then transitions to a temperature learning module, which is composed of an MLP block, a linear layer, and a sigmoid activation function. This module is responsible for generating a scalar temperature parameter, denoted as *T*, which dynamically adjusts the sharpness of the logits used in the knowledge distillation loss calculation. Through this adaptive mechanism, both the student’s and teacher’s logits are softened by T, and subsequently processed through softmax and log-softmax functions to align their distributions.

The final computation of the KL divergence between these adjusted distributions yields the logits-level LKD loss, encapsulated as the mean of KL divergence across the batch. This loss metric serves a pivotal role in guiding the adjustment of the student model’s parameters, ensuring a closer alignment with the teacher model’s output predictions, thereby enhancing the student’s learning trajectory.

**Feature-level LKD loss**. Integral to the LKD framework, the feature-level LKD loss network, illustrated in Fig 2, is designed to critically address and reduce the disparities present in the feature maps between teacher and student models. This network is engineered to amplify the student model’s capacity for learning by imparting the richer, more complex features inherent to the teacher model.

Each set of features, teacher’s (t) and student’s (s), is subjected to mean pooling across the height (*H*) and width (*W*) dimensions, effectively condensing their spatial complexity before further processing through convolutional layers (Conv). These layers are tailored to harmonize the features into a unified representational space, facilitating a meaningful comparison. Subsequently, the congruent features from both the teacher and the student streams are channelled through an additional convolutional layer, where they are further refined. In this stage, the “class id” embeddings are also incorporated, enriching the feature set with class-specific contextual information. Following the convolutional fusion and embedding augmentation, the resulting composite is processed through a convolutional layer and then modulated by a sigmoid function. This sequence yields a dynamic weighting factor, termed ‘weight’, which serves to calibrate the influence of individual feature elements in the loss function. The feature-level LKD loss is thus articulated as the weighted mean squared divergence $(t-s{)}^{2}\times \text{weight}$ between the teacher’s and student’s features. This formulation not only prompts the student model to echo the teacher’s feature representations but also strategically concentrates on elements most germane to effective distillation.

### 4.4 Algorithm analysis: time and memory consumption

**Time consumption**. The LKD method incorporates two primary loops. In the inner loop (Algorithm 1), each iteration performs forward passes through both the teacher and student models, followed by a backward pass to update the student parameters using the LKD loss. The per-iteration cost is similar to standard KD training and scales with the number of student training steps $N_{s}$. In the outer loop (Algorithm 2), after training the student for one epoch, the student model is evaluated on the validation set to compute the CE loss, and gradients with respect to the LKD network parameters θ are computed. This process, repeated for $N_{LKD}$ iterations per epoch, adds an extra overhead. Overall, the time complexity can be approximated as


$$O(N\times (N_{LKD}\times N_{s})),$$

where *N* is the total number of epochs. Careful tuning of $N_{s}$ and $N_{LKD}$ is necessary to balance improved performance with increased training time.

**Memory consumption**. The memory requirements are primarily determined by the storage of the student and teacher models, which are similar to those used in standard KD methods. The additional LKD network, with parameters θ, contributes a relatively modest memory overhead. During the inner loop, intermediate activations and gradients are stored as in conventional backpropagation, while the outer loop may require extra memory for computing gradients with respect to θ. Furthermore, the periodic archiving of student model parameters introduces additional storage costs; however, if managed with a fixed-size buffer or efficient checkpointing, this impact remains limited. Overall, the extra memory overhead due to the LKD components is minor compared to that of the main models.

## 5 Experiments

To evaluate the performance of our LKD, we conduct experiments on two benchmark image classification datasets: ImageNet and CIFAR-100. We use the standard training strategies and model settings following previous KD approaches [16, 28, 43], and detailed settings are provided in S1 and S2 Tables.

### 5.1 Results on ImageNet dataset

The data presented in Table 1 demonstrate the performance metrics of different KD techniques on the ImageNet dataset, using ResNet-34 and ResNet-50 models as teacher networks. For the ResNet-18 (ResNet-34) setting, the LKD technique outperforms all other KD methods with a top-1 accuracy of 72.45% and a top-5 accuracy of 90.61%. This represents an improvement of 2.69% in top-1 accuracy and 1.53% in top-5 accuracy over the stand-alone student model. In the MobileNet (ResNet-50) setting, LKD again achieves the highest performance with a top-1 accuracy of 73.62% and a top-5 accuracy of 91.23%, indicating a 3.49% increase in top-1 and a 1.74% increase in top-5 accuracy over the baseline student model. The outcome underscores the superiority of LKD over conventional and previous state-of-the-art KD techniques, especially in terms of top-1 accuracy.

**Table 1:** Performance on ImageNet dataset. We train the models following the standard training strategy with pre-trained teacher networks ResNet-34 and ResNet-50 provided by Torchvision [42].

Student (teacher)		Tea.	Stu.	KD	Review	DKD	DIST	MSE	LKD
R18 (R34)	Top-1	73.31	69.76	70.66	71.61	71.70	72.07	70.58	72.45
	Top-5	91.42	89.08	89.88	90.51	90.41	90.42	89.95	90.61
MBV1 (R50)	Top-1	76.16	70.13	70.68	72.56	72.05	73.24	72.39	73.62
	Top-5	92.86	89.49	90.30	91.00	91.05	91.12	90.74	91.23

### 5.2 Results on CIFAR datasets

The outcomes for the CIFAR-100 dataset, as detailed in Tables 2 and 3, reveal a comprehensive evaluation of various KD methods across different network architectures. Our LKD outshines previous methods in the majority of evaluated scenarios. In the homogeneous architecture setting, where both teacher and student networks share the same architecture style but differ in size or depth, LKD demonstrates exceptional performance. Specifically, LKD achieves the highest accuracy in all three comparisons, showing improvements with top accuracies of 75.07%, 72.11%, and 76.46% respectively. In the heterogeneous architecture setting, which involves transferring knowledge between different network architectures, LKD again proves to be the most effective method in the majority of cases. For transitions from ResNet-50 to MobileNetV2, ResNet-32x4 to ShuffleNetV1, and ResNet-32x4 to ShuffleNetV2, LKD leads with top accuracies of 69.18%, 76.55%, and 76.92% respectively.

**Table 2:** Results on CIFAR-100 dataset with homogeneous architecture style of teacher and student. The top and bottom model names represent the teacher and student, respectively.

Method	WRN-40-2	ResNet-56	ResNet-32x4
	WRN-40-1	ResNet-20	ResNet-8x4
Teacher	75.61	72.34	79.42
Student	71.98	69.06	72.50
FitNet [44]	72.24 ± 0.24	69.21 ± 0.36	73.50 ± 0.28
RKD [26]	72.22 ± 0.20	69.61 ± 0.06	71.90 ± 0.11
PKT [45]	73.45 ± 0.19	70.34 ± 0.04	73.64 ± 0.18
CRD [28]	74.14 ± 0.22	71.16 ± 0.17	75.51 ± 0.18
KD [6]	73.54 ± 0.20	70.66 ± 0.24	73.33 ± 0.25
DIST [16]	74.73 ± 0.24	71.75 ± 0.30	76.31 ± 0.19
LKD	**75.07** ± 0.22	**72.11** ± 0.17	**76.46** ± 0.28

**Table 3:** Results on CIFAR-100 dataset with heterogeneous architecture style of teacher and student.

Method	ResNet-50	ResNet-32x4	ResNet-32x4
	MobileNetV2	ShuffleNetV1	ShuffleNetV2
Teacher	79.34	79.42	79.42
Student	64.6	70.5	71.82
FitNet [44]	63.16 ± 0.47	73.59 ± 0.15	73.54 ± 0.22
RKD [26]	64.43 ± 0.42	72.28 ± 0.39	73.21 ± 0.28
PKT [45]	66.52 ± 0.33	74.10 ± 0.25	74.69 ± 0.34
CRD [28]	69.11 ± 0.28	75.11 ± 0.32	75.65 ± 0.10
KD [6]	67.35 ± 0.32	74.07 ± 0.19	74.45 ± 0.27
DIST [16]	68.66 ± 0.23	76.34 ± 0.18	**77.35** ± 0.25
LKD	**69.18** ± 0.31	**76.55** ± 0.15	76.92 ± 0.29

### 5.3 Computational time analysis of two-stage optimization

Running the ResNet-20 student model and ResNet-56 teacher model on the CIFAR-100 dataset using an NVIDIA A40 GPU took 1200 seconds longer than the original KD training time of 1 hour, 8 minutes, and 22 seconds. This additional time demonstrates that our bi-level optimization, although slightly increasing computational time, effectively searches for the optimal adaptive LKD loss in an acceptable manner.

### 5.4 Ablation study

**Fig 3:**
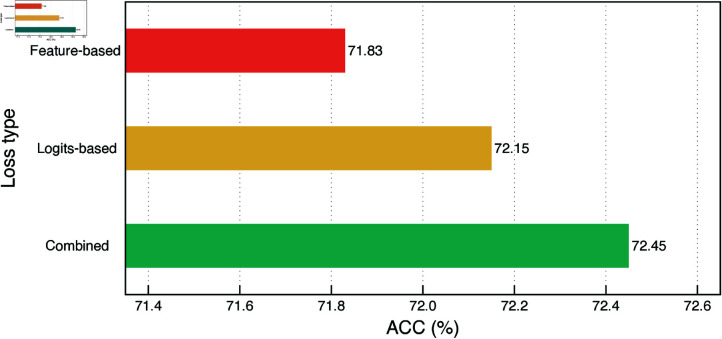
Performance of different distillation loss types.

**Effects of adopting LKD on different types of losses.** In our study, we improved the performance of a ResNet-18 student model by distilling knowledge from a ResNet-34 teacher using different types of loss, as shown in Fig 3. Specifically, using feature-level loss alone led to some improvement, while logit-level loss provided a slight additional boost. The most notable enhancement occurred when we combined both feature-level and logit-level losses in our LKD method. This combination approach significantly outperformed using each loss type separately, highlighting the benefits of integrating multiple loss types for more effective knowledge transfer. This suggests that incorporating both probabilistic and intermediate feature information from the teacher model enables the student model to gain a fuller understanding and achieve better performance.

**Effect of uniform sampling on student model parameters.** As detailed in Table 4, our study investigates the effect of different parameter sampling strategies on KD from ResNet-34 to ResNet-18. We found that uniform sampling increases accuracy by approximately 0.22% over using the latest parameters, demonstrating uniform sampling’s superior effectiveness in optimizing LKD training. This suggests uniform sampling helps prevent potential overfitting associated with training LKD loss using the most recent parameters, thus enhancing the robustness and reliability of the LKD model’s performance.

**Table 4:** Impact of parameter sampling strategies on KD from ResNet-34 to ResNet-18.

Sampling Strategy	Accuracy (%)
Uniform Sampling	72.45
Latest Parameters	72.23

**Effect of the Gaussian sampling of loss training steps.** In our study on LKD training, we compared Gaussian sampling to fixed-step sampling during validation. As shown in Table 5, Our findings reveal that Gaussian sampling, centred around an average of 25 steps, notably increases model accuracy over fixed-step sampling. This indicates the effectiveness of Gaussian sampling in improving LKD training outcomes by introducing beneficial variability. This variability significantly enhances the robustness and stability of the LKD model, leading to better overall performance.

**Table 5:** Comparison of Gaussian sampling and fixed-step sampling in LKD validation.

Sampling Strategy	Accuracy (%)
Fixed-Step Sampling	72.11
Gaussian Sampling	72.45

**Effects of the adopting same batch data.** As illustrated in Fig 1, we consistently apply the same batch data for both the training and validation phases. The evidence presented in Table 6 distinctly demonstrates how strategic data usage significantly affects model performance. The decision to maintain a consistent batch data strategy across both phases culminates in a marked accuracy enhancement of 0.36%, emphatically highlighting the benefits of batch data uniformity within the LKD framework. This finding underscores the importance of input consistency in the LKD learning process. It reveals that for the LKD loss to optimize learning effectively, it must be exposed to the same input that the student model encounters during an epoch iteration. By reviewing the knowledge with identical inputs, the LKD loss can learn more efficiently, indicating the critical role of synchronized data exposure in enhancing the distillation process.

**Table 6:** Impact of batch data consistency in LKD training on ImageNet.

Data Usage Strategy	Accuracy (%)
Same Data	72.45
Distinct Data	72.09

**Static two-stage training vs. dynamic one-stage training.** Delving into the nuances of training methodologies in Section 4.2, our ablation study unveils the inefficiencies tied to static two-stage training approaches. The synthesized results in Table 7 demonstrate a pronounced difference in student model accuracy contingent on the chosen training strategy. A comparative evaluation brings to light a relative accuracy boost of 0.19% favouring the dynamic, integrated training approach, underscoring its definitive advantages in terms of time efficiency and model robustness over the conventional two-stage framework.

**Table 7:** Impact of training approaches on LKD loss and student model accuracy.

Training Approach	Accuracy (%)
Static Two-Stage	72.26
Dynamic One-Stage	72.45

## 6 Conclusion

This study presented the LKD method, a novel framework that autonomously creates adaptive distillation losses, enhancing the KD process beyond traditional, manually crafted, task-specific losses. By employing a bi-level and iterative optimization strategy, LKD aligns distillation losses with student models’ validation loss, utilizing generic loss networks for logits and intermediate features for dynamic optimization. This approach ensures performance improvement and adaptability by adjusting to student models’ evolving states. Incorporating uniform sampling of diverse, previously-trained student models, LKD achieves a more robust loss function, improving distillation efficacy and framework adaptability across various tasks and datasets. Empirical tests on CIFAR and ImageNet demonstrate LKD’s superiority to current KD methods without requiring task-specific adjustments, showcasing its potential for a more adaptive, efficient, and universally applicable knowledge distillation paradigm, poised to advance deep learning and model compression significantly. The implementation code of our LKD framework is publicly available at https://github.com/Ran-Sheng/LKD/tree/main.

## Supporting information

Table S1 Experiment settings for cifar10 (PDF)

Table S2 Experiment settings for cifar100. (PDF)

## References

[1] Dosovitskiy A, Beyer L, Kolesnikov A, Weissenborn D, Zhai X, Unterthiner T. An image is worth 16x16 words: Transformers for image recognition at scale. arXiv, preprint, 2020. https://arxiv.org/abs/2010.11929

[2] Radford A, Narasimhan K, Salimans T, Sutskever I. Improving language understanding by generative pre-training. 2018 (in progress).

[3] Radford A, Wu J, Child R, Luan D, Amodei D, Sutskever I. Language models are unsupervised multitask learners. OpenAI blog. 2019.

[4] Brown T, Mann B, Ryder N, Subbiah M, Kaplan JD, Dhariwal P, et al. Language models are few-shot learners. In: Advances in neural information processing systems 33. 2020, pp. 1877–901.

[5] Han S, Pool J, Tran J, Dally W. Learning both weights and connections for efficient neural network. In: Advances in neural information processing systems 28. 2015.

[6] Hinton G, Vinyals O, Dean J. Distilling the knowledge in a neural network. arXiv, preprint, 2015. arXiv:150302531.

[7] Howard AG, Zhu M, Chen B, Kalenichenko D, Wang W, Weyand T. Mobilenets: efficient convolutional neural networks for mobile vision applications. arXiv, preprint, 2017. https://doi.org/arXiv:170404861

[8] Liu Z, Mu H, Zhang X, Guo Z, Yang X, Cheng KT. Metapruning: meta learning for automatic neural network channel pruning. In: Proceedings of the IEEE/CVF International Conference on Computer Vision, Seoul, Korea (South), 2019. 3296–305.

[9] Renda A, Frankle J, Carbin M. Comparing rewinding and fine-tuning in neural network pruning. arXiv, preprint, 2020. 10.48550/arXiv.2003.02389

[10] Li Y, Gu S, Mayer C, Gool LV, Timofte R. Group sparsity: The hinge between filter pruning and decomposition for network compression. In: Proceedings of the IEEE/CVF conference on computer vision and pattern recognition. arXiv, preprint, 2020, pp. 8018–27.

[11] Zhang Y, Huang T, Liu J, Jiang T, Cheng K, Zhang S. FreeKD: knowledge distillation via semantic frequency prompt. arXiv, preprint, 2023. 10.48550/arXiv.2311.12079

[12] Niu T, Teng Y, Jin L, Zou P, Liu Y. Pruning-and-distillation: one-stage joint compression framework for CNNs via clustering. Image Vis Comput. 2023;136:104743. doi: 10.1016/j.imavis.2023.104743

[13] Karim AAJ, Asad KHM, Alam MGR. Larger models yield better results? Streamlined severity classification of ADHD-related concerns using BERT-based knowledge distillation. PLoS One. 2025;20(2):e0315829. doi: 10.1371/journal.pone.0315829 39913350 PMC11801592

[14] Pavel MA, Islam R, Babor SB, Mehadi R, Khan R. Non-small cell lung cancer detection through knowledge distillation approach with teaching assistant. PLoS One. 2024;19(11):e0306441. doi: 10.1371/journal.pone.0306441 39504338 PMC11540227

[15] Zagoruyko S, Komodakis N. Paying more attention to attention: Improving the performance of convolutional neural networks via attention transfer. arXiv, preprint, 2016. https://doi.org/arXiv:161203928

[16] Huang T, You S, Wang F, Qian C, Xu C. Knowledge distillation from a stronger teacher. In: Advances in Neural Information Processing Systems, 35. 2022, pp. 33716–27.

[17] Adriana R, Nicolas B, Ebrahimi KS, Antoine C, Carlo G, Yoshua B. Fitnets: hints for thin deep nets. In: Proceedings of the International Conference on Learning Representations (ICLR). arXiv, preprint, 2015, p. 3.

[18] Komodakis N, Zagoruyko S. Paying more attention to attention: improving the performance of convolutional neural networks via attention transfer. In: Proceedings of the International Conference on Learning Representations (ICLR). arXiv, preprint, 2017.

[19] Huang Z, Wang N. Like what you like: Knowledge distill via neuron selectivity transfer. arXiv, preprint, 2017. https://arxiv.org/abs/1707.01219

[20] Yim J, Joo D, Bae J, Kim J. A gift from knowledge distillation: Fast optimization, network minimization and transfer learning. In: 2017 IEEE Conference on Computer Vision and Pattern Recognition (CVPR), Honolulu, HI, USA, 2017, pp. 4133–41.

[21] Passalis N, Tefas A. Probabilistic knowledge transfer for deep representation learning. CoRR. 2018;1(2):5. doi: abs/180310837 10.1109/TNNLS.2020.2995884 32479404

[22] Kim J, Park S, Kwak N. Paraphrasing complex network: network compression via factor transfer. In: Advances in Neural Information Processing Systems 31. 2018.

[23] Tung F, Mori G. Similarity-preserving knowledge distillation. In: 2019 IEEE/CVF International Conference on Computer Vision (ICCV), Seoul, Korea (South), 2019, pp. 1365–74. 10.1109/ICCV.2019.00145

[24] Peng B, Jin X, Liu J, Li D, Wu Y, Liu Y, et al. Correlation congruence for knowledge distillation. In: Proceedings of the IEEE/CVF International Conference on Computer Vision, 2019, pp. 5007–5016.

[25] Ahn S, Hu SX, Damianou A, Lawrence ND, Dai Z. Variational information distillation for knowledge transfer. In: Proceedings of the IEEE/CVF conference on computer vision and pattern recognition, 2019, pp. 9163–71.

[26] Park W, Kim D, Lu Y, Cho M. Relational knowledge distillation. In: Proceedings of the IEEE/CVF conference on computer vision and pattern recognition, 2019, pp. 3967–76.

[27] Heo B, Lee M, Yun S, Choi JY. Knowledge transfer via distillation of activation boundaries formed by hidden neurons. In: Proceedings of the AAAI Conference on Artificial Intelligence, vol. 33. 2019, pp. 3779–87. 10.1609/aaai.v33i01.33013779

[28] Tian Y, Krishnan D, Isola P. Contrastive representation distillation. arXiv, preprint, 2019. https://arxiv.org/abs/1910.10699

[29] Mirzadeh SI, Farajtabar M, Li A, Levine N, Matsukawa A, Ghasemzadeh H. Improved knowledge distillation via teacher assistant. In: Proceedings of the AAAI conference on artificial intelligence, vol. 34. 2020, pp. 5191–8. 10.1609/aaai.v34i04.5963

[30] Son W, Na J, Choi J, Hwang W. Densely guided knowledge distillation using multiple teacher assistants. In: Proceedings of the IEEE/CVF International Conference on Computer Vision. 2021, pp. 9395–404.

[31] Park DY, Cha MH, Kim D, Han B. Learning student-friendly teacher networks for knowledge distillation. In: Advances in neural information processing systems 34. 2021, pp.13292–303.

[32] Shao B, Chen Y. Multi-granularity for knowledge distillation. Image Vis Comput. 2021;115:104286. doi: 10.1016/j.imavis.2021.104286

[33] Tang Y, Chen Y, Xie L. Self-knowledge distillation based on knowledge transfer from soft to hard examples. Image Vis Comput. 2023;135:104700. doi: 10.1016/j.imavis.2023.104700

[34] Guo J. Reducing the teacher-student gap via adaptive temperatures. 2022. https://openreview.net/forum?id=h-z_zqT2yJU

[35] Liu J, Liu B, Li H, Liu Y. Meta knowledge distillation. arXiv, preprint, 2022. 10.48550/arXiv.2202.07940

[36] Zheng K, Yang EH. Knowledge distillation based on transformed teacher matching. arXiv, preprint, 2024. https://doi.org/arXiv:240211148

[37] Yu L, Li Y, Weng S, Tian H, Liu J. Adaptive multi-teacher softened relational knowledge distillation framework for payload mismatch in image steganalysis. J Vis Commun Image Represent. 2023;95:103900. doi: 10.1016/j.jvcir.2023.103900

[38] Yang S, Xu L, Ren J, Yang J, Huang Z, Gong Z. Adaptive temperature distillation method for mining hard sample’s knowledge. 10.2139/ssrn.4466292

[39] Huang T, Li Z, Lu H, Shan Y, Yang S, Feng Y, et al. Relational surrogate loss learning. In: International Conference on Learning Representations. 2022. Available from: https://openreview.net/forum?id=dZPgfwaTaXv

[40] Anandalingam G, Friesz TL. Hierarchical optimization: an introduction. Ann Oper Res. 1992;34(1):1–11. doi: 10.1007/bf02098169

[41] Colson B, Marcotte P, Savard G. An overview of bilevel optimization. Ann Oper Res. 2007;153(1):235–56. doi: 10.1007/s10479-007-0176-2

[42] Marcel S, Rodriguez Y. Torchvision the machine-vision package of torch. In: Proceedings of the 18th ACM International Conference on Multimedia, 2010, pp. 1485–8.

[43] Huang T, Zhang Y, Zheng M, You S, Wang F, Qian C. Knowledge diffusion for distillation. In: Advances in Neural Information Processing Systems 36. 2024.

[44] Romero A, Ballas N, Kahou SE, Chassang A, Gatta C, Bengio Y. Fitnets: hints for thin deep nets. arXiv, preprint, 2014. https://doi.org/arXiv:1412.6550

[45] Passalis N, Tzelepi M, Tefas A. Probabilistic knowledge transfer for lightweight deep representation learning. IEEE Trans Neural Netw Learn Syst. 2021;32(5):2030–9. doi: 10.1109/TNNLS.2020.2995884 32479404

