# Supplementary material for: Tailored knowledge distillation with automated loss function learning
Source: PLoS One. 2025 Jun 11;20(6):e0325599. doi: 10.1371/journal.pone.0325599 (PMC12157245; doi:10.1371/journal.pone.0325599)
Supplement: Table S1 — (PDF) [file pone.0325599.s001.pdf]

Table S1: **Experiment settings for cifar10**

Parameter	Value
**Dataset**	cifar10
**aa**	null
**Batch Size**	128
**Color Jitter**	0.0
**Cutout Length**	16
**Decay by Epoch**	false
**Decay Epochs**	3
**Decay Rate**	0.967
**Drop**	0.0
**Epochs**	600
**Log Interval**	50
**Learning Rate (lr)**	0.1
**Smoothing**	0.0
**Min LR**	1.0×10^{-6}
**Model EMA**	false
**Model EMA Decay**	0.9998
**KD Loss EMA Decay**	0.99
**Momentum**	0.9
**Optimizer (opt)**	sgd
**Opt Betas**	null
**Opt Eps**	1.0×10^{-8}
**Remode**	const
**Reprob**	0.0
**Scheduler (sched)**	cosine
**Seed**	42
**Warmup Epochs**	0
**Warmup LR**	0.2
**Weight Decay**	1.0×10^{-4}
**Workers**	4
**SGD No Nesterov**	True
**Opt No Filter**	True
**Clip Grad Max Norm**	1.0
**dyrep Settings**	
**dyrep**	False
**dyrep Adjust Interval**	15
**dyrep Recal BN Every Epoch**	False
**KD Settings**	
**Ori Loss Weight**	1.0
**KD Loss Weight**	1.0
**KD**	learnable_kd
**Teacher Model**	cifar_vgg16
**Teacher Pretrained**	True
**Teacher Checkpoint**	experiments/vgg16_cifar10_exp/best.pth.tar
**Model Config**	configs/models/VGG/vgg16_half_cifar10.yaml
**Experiment**	learnable_kd_loss_cifar10
**KD Optimizer (kd_opt)**	sgd
**KD LR**	1×10^{-3}
**KD Opt Eps**	1.0×10^{-8}
**KD Momentum**	0.9
**KD Weight Decay**	1×10^{-4}
**Update Student Params Steps**	50
