# Supplementary material for: Tailored knowledge distillation with automated loss function learning
Source: PLoS One. 2025 Jun 11;20(6):e0325599. doi: 10.1371/journal.pone.0325599 (PMC12157245; doi:10.1371/journal.pone.0325599)
Supplement: Table S2 — (PDF) [file pone.0325599.s002.pdf]

Table S2: **Experiment settings for cifar100**

Parameter	Value
**Dataset**	cifar100
**Image Mean**	[0.5071, 0.4867, 0.4408]
**Image Std**	[0.2675, 0.2565, 0.2761]
**aa**	null
**Batch Size**	64
**Color Jitter**	0.0
**Cutout Length**	0
**Decay by Epoch**	True
**Decay Epochs**	30
**Decay Rate**	0.1
**Drop**	0.0
**Epochs**	240
**Log Interval**	50
**Learning Rate (lr)**	0.05
**Smoothing**	0.0
**Min LR**	1.0×10^{-6}
**Model EMA**	false
**Model EMA Decay**	0.9998
**KD Loss EMA**	false
**KD Loss EMA Decay**	0.99
**Momentum**	0.9
**Optimizer (opt)**	sgd
**Opt Betas**	null
**Opt Eps**	1.0×10^{-8}
**Remode**	const
**Reprob**	0.0
**Scheduler (sched)**	step
**Seed**	42
**Warmup Epochs**	120
**Warmup LR**	0.05
**Weight Decay**	5.0×10^{-4}
**Workers**	4
**SGD No Nesterov**	True
**Opt No Filter**	True
**KD Settings**	
**Ori Loss Weight**	1.0
**KD Loss Weight**	1.0
**KD**	learnable_kd
**Teacher Model**	cifar_resnet56
**Teacher Pretrained**	True
**Teacher Checkpoint**	./data/saved$_{c}kpts/resnet56_vanilla/ckpt_epoch_240.pth$
**Model**	cifar_resnet20
**Experiment**	1_LKD_cifar100
**KD Optimizer (kd_opt)**	adamw
**KD LR**	0.001
**KD Opt Eps**	1.0×10^{-8}
**KD Momentum**	0.9
**KD Weight Decay**	1×10^{-4}
**Update Student Params Steps**	50
